# Co-Administration of Iron and a Bioavailable Curcumin Supplement Increases Serum BDNF Levels in Healthy Adults

**DOI:** 10.3390/antiox9080645

**Published:** 2020-07-22

**Authors:** Helena Tiekou Lorinczova, Owen Fitzsimons, Leah Mursaleen, Derek Renshaw, Gulshanara Begum, Mohammed Gulrez Zariwala

**Affiliations:** 1 School of Life Sciences, University of Westminster, 115 New Cavendish Street, London W1W 6UW, UK; H.Lorinczova@westminster.ac.uk (H.T.L.); O.Fitzsimons@westminster.ac.uk (O.F.); w1655446@my.westminster.ac.uk (L.M.); begumru@westminster.ac.uk (G.B.); 2 The Cure Parkinson’s Trust, 120 New Cavendish St, Fitzrovia, London W1W 6XX, UK; 3 Centre for Sport, Exercise and Life Sciences, Faculty of Health and Life Sciences, Coventry University, Priory St, Coventry CV1 5FB, UK; derek.renshaw@coventry.ac.uk

**Keywords:** BDNF (brain-derived neurotrophic factor), curcumin, iron, ferrous sulphate, supplementation, brain function, antioxidant capability

## Abstract

Brain-derived neurotrophic factor (BDNF) is key for the maintenance of normal neuronal function and energy homeostasis and has been suggested to improve cognitive function, including learning and memory. Iron and the antioxidant curcumin have been shown to influence BDNF homeostasis. This 6-week, double blind, randomized, placebo-controlled study examined the effects of oral iron supplementation at low (18 mg) and high (65 mg) ferrous (FS) iron dosages, compared to a combination of these iron doses with a bioavailable formulated form of curcumin (HydroCurc^TM^; 500 mg) on BDNF levels in a healthy adult cohort of 155 male (26.42 years ± 0.55) and female (25.82 years ± 0.54) participants. Participants were randomly allocated to five different treatment groups: both iron and curcumin placebo (FS0+Plac), low dose iron and curcumin placebo (FS18+Plac), low dose iron and curcumin (FS18+Curc), high dose iron and curcumin placebo (FS65+Plac) and high dose iron and curcumin (FS65+Curc). Results showed a significant increase in BDNF over time (26%) in the FS18+Curc group (*p* = 0.024), and at end-point between FS18+Curc and FS18+Plac groups (35%, *p* = 0.042), demonstrating for the first time that the combination with curcumin, rather than iron supplementation alone, results in increased serum BDNF. The addition of curcumin to iron supplementation may therefore provide a novel approach to further enhance the benefits associated with increased BDNF levels.

## 1. Introduction

Iron is a critical micronutrient vital for oxygen transport and energy production via cellular respiration [1] as well as for the development and maintenance of normal neuronal function [2]. Assuming a mixed human diet, dietary iron intake ranges from 12 to 18 mg/day, of which 1–2 mg is absorbed into the circulation via the duodenum and proximal jejunum [3,4]. Iron intake often does not meet the body’s requirements due to inadequate nutrient intake and poor bioavailability on account of the complexities of iron absorption (such as the influence of dietary iron inhibitors and enhancers), thus leading to widespread iron deficiency [5,6,7]. This is a worldwide public health issue as iron deficiency has been attributed to more than 60% of anaemia cases, a condition estimated to affect around two billion people globally [8,9,10,11].

Iron deficiency can lead to impaired cognitive and physical development in children, compromise physical and cognitive performance in adults [12,13,14,15] and has been linked with fatigue [16], impaired quality of life [17] and reduced mood [14,15]. In addition to being a critical component of haemoglobin [1], iron also has a key role in electron transport during cellular respiration [18], deoxyribonucleic acid (DNA) synthesis [19] and is an important cofactor in the synthesis of neurotransmitters such as serotonin and norepinephrine [20].

Hippocampal brain-derived neurotrophic factor (BDNF) is a neurotrophic growth factor, which is suggested to be essential for normal neuronal development and cognitive function [21]. BDNF expression is associated with neurotransmitter levels and its synthesis is activated by neuronal activity and by increased cytoplasmic calcium levels via the activation of the transcription factor cyclic adenosine monophosphate (cAMP) responsive element binding protein (CREB) [22,23]. As well as being essential for maintaining the normal function of mature neurons, BDNF also regulates dendrite growth, spinal development and aids long term synaptic potentiation during neuronal development, which is associated with learning and memory formation [21]. BDNF exhibits neuroprotective effects via its role in the modulation of synaptic plasticity and function [24] and has been found to enhance explicit memory encoding, storage and retrieval of information in the hippocampal region of the brain [25]. In addition to its role in cognitive function, BDNF is thought to be a major contributor to energy homeostasis [26]. Therefore, it is thought that increased levels of BDNF could enhance cognitive capacity and potentially lead to reduced fatigue [24]. It has been shown that radiolabelled BDNF rapidly crosses the blood-brain barrier (BBB) and the efflux of unmodified BDNF to the circulation following intracerebroventricular injection has also been demonstrated in mice [27]. Further animal studies have also demonstrated the influx of BDNF from the circulation to the brain and vice versa and suggested that this may occur via a saturable transport mechanism [28,29]. Furthermore, positive correlations and parallel changes between peripheral and central BDNF levels have been shown in animal studies, indicating that circulatory BDNF measured in plasma or serum can be used as a biomarker for central BDNF levels [29,30,31].

Although the mechanistic pathways of iron and BDNF interaction are not yet fully elucidated, optimal iron levels are thought to be essential for BDNF homeostasis [32]. It has been suggested that when brain iron levels are low, BDNF may be down regulated as a consequence of altered neurotransmitter levels [33]. However, excessive cerebral iron levels can also reduce BDNF expression and are associated with cognitive and mental impairments [34,35,36]. This is claimed to be a consequence of the brain’s altered redox state, as high iron levels facilitate the production of detrimental reactive oxygen species (ROS) [36,37], and the brain lacks efficient levels of antioxidant defences to counteract them [35,36]. The accumulation of ROS results in oxidative stress, damage to DNA, proteins and lipids, which can result in cell death [38,39]. Antioxidants can counteract the effects of ROS and have therefore generated interest as molecules that could potentially enhance BDNF levels [40,41].

Curcumin, a non-flavonoid polyphenol, is the most biologically active antioxidant component in the rhizomatous spice *Curcuma longa* Linnaeus (L.) or turmeric [42]. Curcumin is a pleiotropic compound with wide ranging beneficial properties including antioxidative, anti-inflammatory and neuroprotective effects [43,44]. Curcumin and its analogue curcuminoids, demethoxycurcumin (DMC) and bisdemethoxycurcumin (BDMC), can exist in two different forms depending on their environment; keto and enol [45]. In their enol form, they are capable of accepting as well as donating hydrogen and have metal chelation characteristics [46]. Curcumin is extensively studied for its ROS scavenging properties [47,48,49,50]. It acts as a free-radical chain breaker, capable of donating hydrogen to ROS due to the presence of a hydroxyl group in its structure [51]. Furthermore, studies examining the neuroprotective properties of curcumin have shown that it has potential as a treatment for neurological disorders, such as depression, bipolar disorders and neurodegenerative diseases [44,52]. Since curcumin is lipophilic in nature, it is assumed to be able to cross the BBB [53]. However, it is debated whether it can access the brain at therapeutic concentrations as it is rapidly metabolised following ingestion [54]. The pharmacological use of curcumin has therefore been limited due to its poor bioavailability, limited bio-distribution, poor stability and short half-life [55]. Formulation science strategies have shown that nanocarrier delivery systems such as liposomes and micelles may address these limitations and enhance curcumin’s therapeutic potential [44,56,57]. A recent study by Briskey and colleagues [58] demonstrated that formulation of curcumin using a novel delivery system comprising a mixture of surfactants, polar lipids and solvents (known as LipiSperse^®^) significantly increases the plasma concentration of curcumin in human volunteers, further highlighting the application of delivery systems to increase the bioavailability of poorly absorbed molecules.

As iron and curcumin have both been independently associated with BDNF homeostasis in animal and cellular models [33,34,35,36,52], there is scope to further investigate their co-administration. The aim of this 6-week, double blind, randomized, placebo-controlled study was to examine the effects of oral iron supplementation at low (18 mg) versus high (65 mg) ferrous iron dosages, either alone or co-administered with curcumin (500 mg) supplementation on serum BDNF levels in healthy adults. The study was designed to determine whether co-administration of ferrous sulphate and a bioavailable formulated curcumin supplement (commercially available as HydroCurc™) would amplify serum levels of BDNF.

## 2. Materials and Methods

### 2.1. Study Design

The present double blind, placebo-controlled, randomized study recruited 155 healthy participants (79 males and 76 females) for a study duration of 6 weeks. Sample size was calculated using G*Power statistical analysis software [59,60] to achieve 80% power.

**Figure 1:**
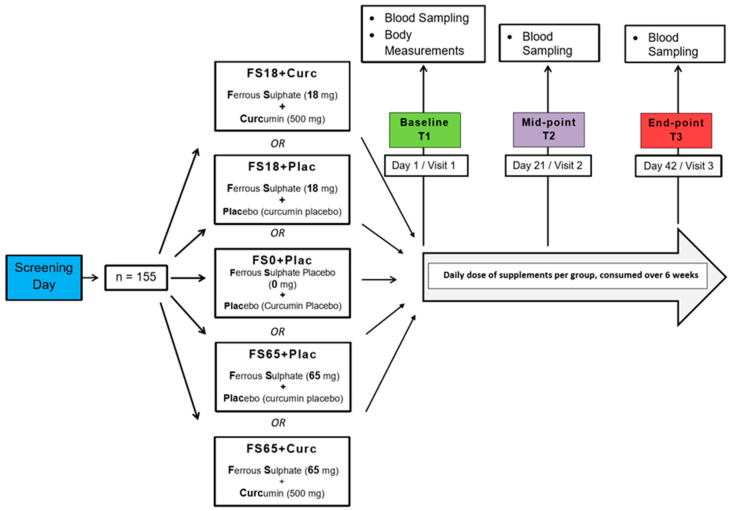
Study design. Participants who met the inclusion criteria during the screening day were randomly assigned to 5 different treatment groups (*n* = 31/group). There were three visit days over the study duration of 6 weeks. On the first visit day, (Baseline) body measurements, blood samples and questionnaires were collected from the participants. On the following visit days, (Mid-point and End-point) blood samples and questionnaires were collected from the participants.

Study participants were randomly allocated to one of five different treatment groups using the online service by Study Randomizer [61], via a permuted block and gender-balanced randomisation algorithm with 31 participants in each group. The 5 different treatment groups were ferrous sulphate and curcumin placebos (FS0+Plac), low ferrous sulphate (18 mg elemental iron) and curcumin placebo (FS18+Plac), low ferrous sulphate (18 mg elemental iron) and 500 mg curcumin (FS18+Curc), high ferrous sulphate (65 mg elemental iron) and curcumin placebo (FS65+Plac) and high ferrous sulphate (65 mg elemental iron) and 500 mg curcumin (FS65+Curc) (Figure 1).

Healthy adults aged between 18 and 40 years with ferritin levels in the normal physiological range were recruited into the study. Normal ferritin was defined as 15–300 µg/L for men and 15–200 µg/L for women, according to United Kingdom (UK) guidelines [62,63]. Any participants with haemoglobin values below the World Health Organization (WHO) cut-off for anaemia [64] were not enrolled in the study (<130 g/L for men and <120 g/L for women). For the purpose of sub-analysis, participants (within their allocated treatment groups), were categorised according to baseline ferritin levels; <50 µg/L was classified as ‘low’ and ≥50 µg/L as ‘normal’ ferritin values [65,66,67,68,69].

On the screening day, participants were assessed to ensure they complied with the inclusion/exclusion criteria by means of a comprehensive interview. Exclusion criteria included the following: any diagnosis of medical conditions or comorbidities, currently trying to conceive, pregnancy or lactating, and/or any chronic menstrual disorders or menopausal changes. Furthermore, those who had issues related to oral supplement ingestion, were on any medication or supplementation, exceeded 21 units/week of alcohol consumption, experienced any chronic gastrointestinal problems, eating disorders, psychological conditions or presented with hypo/hypertensive blood pressure, were excluded from study [70,71].

Participants signed written informed consent and ethical approval (ID: ETH1718-0907) was granted by the Faculty of Science and Technology Ethics Committee, University of Westminster, in accordance with the ethical standards of the Helsinki Declaration of 1975.

### 2.2. Supplementation

As per Figure 1, participants were provided two different doses (high and low) of ferrous sulphate supplement, co-administered with a dose of curcumin or equivalent placebo(s) (depending on supplement group allocation). The high dose ferrous sulphate supplementation (200 mg/day, 65 mg elemental iron) is based upon the traditional first line oral iron therapy for treatment and prophylaxis of iron deficiency and iron deficiency anaemia [72,73]. The low dose ferrous sulphate supplementation (55 mg/day with 18 mg elemental iron) is based upon the recommended Daily Value (DV) of iron as per the Food and Drug Administration (FDA) [74]. Curcumin supplements were in the form of 500 mg/day of formulated curcumin (HydroCurc™, Pharmako Biotechnologies Pty Ltd. New South Wales, Australia). This formulation consisted of 85% total curcuminoids (80% curcumin, 17% DMC and 3% BDMC) entrapped in a proprietary delivery system (LipiSperse^®^, Pharmako Biotechnologies Pty Ltd. New South Wales, Australia) that has previously been shown to have enhanced bioavailability and deliver a higher therapeutic curcumin dose [58]. Microcrystalline cellulose served as a placebo as well as the bulking agent in the capsules of active ingredients. White-opaque hydroxypropyl methylcellulose (HPMC) capsules were used, with sizes of #1 and #00, for the ferrous sulphate and curcumin supplements, respectively. The supplements were presented in white, screw lid bottles, labelled with the related group codes. The participants were required to take one ferrous sulphate and one curcumin supplement per day with water, at least 2 h after or 1 h before food consumption; at separate times.

### 2.3. Physical Examination

Baseline anthropometric measurements were collected by trained research staff. Height was measured using a Seca 287 ultrasonic stadiometer (Seca GmbH & Co. KG, Hamburg, Germany). Weight and body mass index (BMI), were measured using the Seca 515 medical Body Composition Analyser (Seca GmbH & Co. KG, Hamburg, Germany). Blood pressure (BP) was measured using an Omron M6 BP monitor (Omron, Hoofddorp, The Netherlands).

### 2.4. Blood Collection

Participants attended blood collection appointments following an overnight fast (12 h fast). Venous blood samples were collected at baseline, mid-point (21 day) and end-point (42 day) visits from the antecubital fossa by venepuncture (using a 21G needle). Approximately 10 mL of blood was collected from each participant per procedure using Becton Dickinson (BD) Vacutainer ^®^ serum-separating tubes (SST) (BD, Oxford, UK). Blood in the SST was left to coagulate at room temperature for 45 min and then centrifuged (Hettich 340r, Hettich GmbH & Co. KG, Tuttlingen, Germany) for 10 min at 3857 g. Serum supernatant was aliquoted into 1.5 mL microcentrifuge tubes post-centrifugation and stored at −80 °C.

### 2.5. Ferritin Assay

Serum ferritin samples were analysed using a Horiba ABX Pentra 400 (Horiba Ltd., Kyoto, Japan) multiparametric medical bench top chemistry analyser, compliant with the National Committee for Clinical Laboratory Standards (NCCLS) [75]. Ferritin values were determined by latex-enhanced immunoturbidimetric assay, in accordance with the manufacturer’s protocol and as previously described by Simó et al. [76].

### 2.6. BDNF Assay

Serum was assayed for BDNF levels using the Biosensis Mature BDNF Rapid^TM^ enzyme-linked immunosorbent assay (ELISA) Kit (ATI Atlas, Chichester, UK) following the manufacturer’s protocol, using a dilution factor of 1:100. Pre-coated microplates were incubated with 100 µL of diluted BDNF standards, quality control (QC) samples, serum samples (1:100) or blanks (sample diluent only) for 45 min on a plate shaker (140 rpm), at room temperature (RT). Plates were then washed five times with wash buffer (200 µL per well). After the addition of 100 µL detection antibody per well, the plates were incubated on the plate shaker (140 rpm at RT) for 30 min. Following five more washes, 100 µL of 1× streptavidin-HRP conjugate was added to each well. The plates were incubated for a further 30 min at 140 rpm (RT). The plates were then washed 5 times and 100 µL of 3,3′,5,5′-Tetramethylbenzidine (TMB) was added to each well and incubated at RT for 9 min in the dark before the addition of 100 µL of stop solution into each well. The absorbance was read with a SPECTROstar^®^ Nano microplate reader (BMG Labtech GmbH, Ortenberg, Germany) at 450 nm (within 5 min).

### 2.7. Study Compliance

Compliance with the study protocol (including supplementation) and adverse effects were checked during the mid-point (week 3) and at the end-point (week 6) of each study period and any deviation from the study protocol was noted and assessed. Adherence to daily supplementation of the active or placebo capsules was ≥80% at all times. Data from all participants who successfully completed the study period was utilised for further analysis. 

### 2.8. Statistical Analysis

Values are expressed as mean ± Standard Error of Mean (SEM). The BDNF assay results were statistically analysed using a two-way, repeated measures analysis of variance (ANOVA) or mixed effects model (where missing values were present). Post-hoc tests (Sidak’s and Tukey’s) were carried out to assess differences between and within treatment groups (PRISM software package, Version 8, Graphpad Software Inc., San Diego, CA, USA).

## 3. Results

Of the 155 participants recruited, 150 completed all study visits. Two participants withdrew from the FS0+Plac groups, one from nausea after the baseline visit and one from loss of interest in the study after the mid-point visit. One participant also withdrew from the FS65+Curc group after the baseline visit due to loss of interest and another withdrew due to gastric distress. A participant in the FS18+Plac group was excluded from the study due to incomplete blood sampling at the mid-point (Figure S1).

The mean age of participants was 26.12 years (±0.39). There was no significant difference in mean age between the five treatment groups. There were also no significant differences observed in anthropometric measurements of participants (Table 1). The study population was of mixed ethnicity, representative of the population at the site of recruitment (London, UK).

At baseline, no significant differences were observed in mean ferritin levels across the treatment groups (Table 2). However, there was a significant difference observed in baseline BDNF levels between the FS18+Plac (37.16 ng/mL) and FS18+Curc (30.28 ng/mL) groups, with the mean BDNF being (22.7% *p* = 0.049) higher in the FS18+Plac group compared to the FS18+Curc group (Table 2). No significant differences in baseline BDNF were observed when comparing any of the other groups (Table 2).

**Figure 2:**
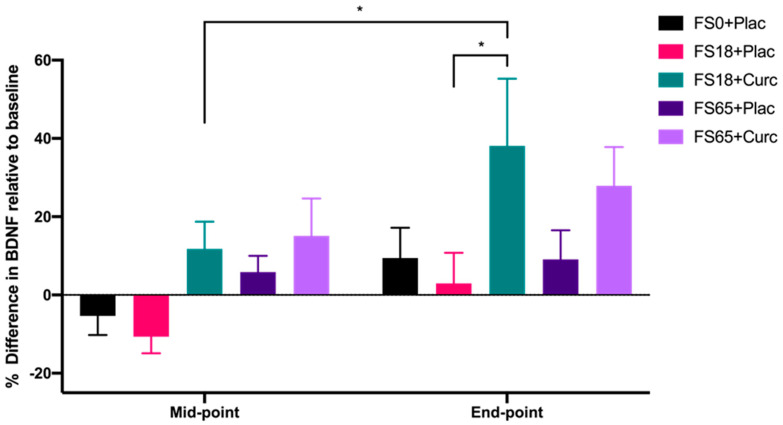
BDNF levels are expressed as percentage difference compared to baseline levels (mean ± SEM): FS0+Plac [Mid-point *n* = 29; End-point *n* = 28], FS18+Plac [Mid-point *n* = 30; End-point *n* = 29], FS18 + Curc [Mid-point *n* = 31; End-point *n* = 31], FS65+Plac [Mid-point *n* = 30; End-point *n* = 30] and FS65 + Curc [Mid-point *n* = 29; End-point *n* = 29]). Samples were collected and analysed at mid-point (day 21) and end-point (day 42). * represents significance values when comparing each condition and time points within the same condition. (* *p* < 0.05).

When evaluating the effect of treatment group on serum BDNF levels after 21 day (mid-point) and 42 day (end-point) supplementation, significant differences were observed between the different treatment groups (F(4, 144) = 2.746, *p* = 0.031) and the two time points (F(1, 142) = 11.36, *p* = 0.001). A significant increase of 26.34% in BDNF levels from mid-point to end-point was observed in participants taking FS18+Curc (*p* = 0.024) (Figure 2). At the end-point, there was also a significant difference observed in BDNF levels between the FS18+Curc and FS18+Plac groups (*p* = 0.042), with the FS18+Curc treatment resulting in a 34.94% higher concentration of BDNF than FS18+Plac (Figure 2). A similar trend of increased BDNF was observed at the end-point when comparing the FS65+Curc treatment with FS65+Plac, however this was not significant (Figure 2). There were no other significant differences in BDNF levels between or within treatment groups (Figure 2).

**Figure 3:**
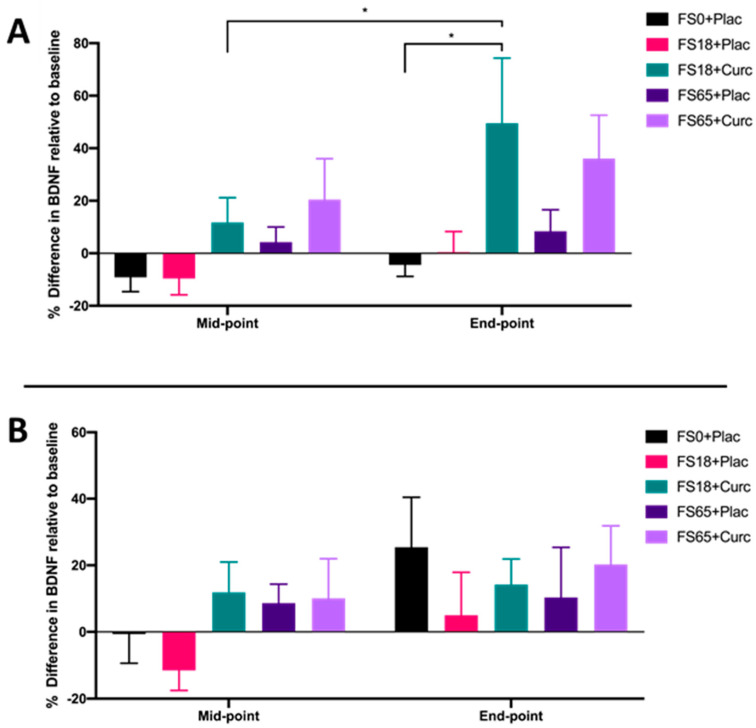
(**A**). Mean ± SEM, % difference in BDNF for participants with low ferritin at baseline (< 50 µg/L): FS0+Plac [Mid-point *n* = 16; End-point: *n* = 15]; FS18+Plac [Mid-point *n* = 14; End-point *n* = 13]; FS18+Curc [Mid-point *n* = 21; End-point *n* = 21]; FS65+Plac [Mid-point *n* = 19; End-point *n* = 19] and FS65+Curc [Mid-point *n* = 14; End-point *n* = 14]. Samples were collected and analysed at mid-point (day 21) and end-point (day 42). (**B**). Mean ± SEM, % difference in BDNF for participants with normal ferritin (≥ 50 µg/L): FS0+Plac [Mid and End-point *n* = 15]; FS18+Plac [Mid and End-point *n* = 16]; FS18+Curc [Mid and End-point *n* = 10]; FS65+Plac [Mid and End-point *n* = 11] and FS65+Curc [Mid and End-point *n* = 15]. * represents significance values when comparing each condition and time points within the same condition. (* *p* < 0.05).

When participants were sub-grouped according to low ferritin (<50 µg/L) and normal ferritin (≥50 µg/L) values, a significant increase in BDNF from mid-point to end-point was observed in the cohort with low ferritin who were supplemented with FS18+Curc (*p* = 0.019) (Figure 3A). Although no significant difference in BDNF was observed between the FS18+Curc and FS18+Plac group for participants with low baseline ferritin, the FS18+Curc group had significantly higher BDNF at the end-point compared to the FS0+Plac group, (increased by 53.78%, *p* = 0.028) (Figure 3A). No significant differences in BDNF levels were observed between treatment groups, at either time point, in participants with normal ferritin levels (Figure 3B).

No significant differences were observed in baseline ferritin levels between the low and normal ferritin groups (Table 3 and Table 4). In the low ferritin sub-group (Table 3), significant differences were observed between baseline, mid-point and end-point time points (F (2, 158) = 27.81, *p* < 0.0001). A significant increase of 35.17% in ferritin levels was observed at end-point compared to baseline in the FS18+Curc group (*p* = 0.0013) (Table 3). A significant increase of 58.75% in ferritin levels was also observed at end-point compared to baseline in the FS65+Curc group (*p* = 0.0002) (Table 3).

Furthermore, there were significant increases from baseline for the FS65+Plac group at mid-point (43.6%) and end-point (68.34%) (*p* = 0.0014 and *p* < 0.0001, respectively) (Table 3). In the normal ferritin sub-group, there was no significant effect observed in ferritin levels over time or between groups in relation to any supplementation (Table 4).

## 4. Discussion

Research has shown BDNF to have a key role in the maintenance of normal neuronal function [21], energy homeostasis [26] and, in animal studies, it has been demonstrated to enhance declarative memory in the developed nervous system [25]. These findings indicate that increased BDNF levels could help to improve cognitive function. Iron supplementation in mice [32] and formulated curcumin supplementation in humans have been shown to independently increase BDNF levels [52], indicating that the combination of these two supplements could possibly express an additive effect on serum BDNF levels. The current study therefore aimed to examine the effects of iron supplement dose in the presence or absence of formulated curcumin on BDNF levels in a healthy adult population aged 18 to 40 years.

Study compliance was high with approximately 3% of initial recruits dropping out or being excluded. Although gastrointestinal effects (such as nausea or delayed bowel movements) were reported by some participants, such incidences were usually mild or transient. High compliance and mild, transient incidences seen in the current study, could be attributed to the dosages of ferrous sulphate being within ranges that are unlikely to cause a high degree of gastric distress, which often results in the poor compliance typically seen in oral iron supplementation [77].

All participants had ferritin values in the normal range for the UK (15–300 µg/L—men and 15–200 µg/L—women) [63]. The 2011 WHO guidelines state that serum ferritin less than 15 µg/L is a specific indicator for iron deficiency [78]. Globally, there is ongoing debate calling for an increase in the normal ferritin cut-off value. Numerous authors [79,80,81] recommend a serum ferritin cut-off of 30 µg/L, as it has been shown to be indicative of insufficient iron stores. Iron therapy may therefore be appropriate in such individuals (ferritin <30 µg/L) to support erythropoiesis as well as normal physical performance and cognitive health [80]. It has also been argued that ferritin below 50 µg/L may indicate reduced bone marrow iron stores or latent iron deficiency [81]. Furthermore, a reduction in fatigue has been reported following iron supplementation in people with ferritin below 50 µg/L [66,68], suggesting the potential therapeutic value of iron supplementation within this ferritin range.

Similar to Verdon et al. [66] and Vaucher et al. [68], whilst taking into account the recent work from Soppi [81], data from the current study were sub-grouped into ‘low’ (<50 µg/L) ferritin and ‘normal’ (≥50 µg/L) ferritin participants (Figure 3A,B, respectively). Participants with serum ferritin below 50 µg/L showed the same trend (increased levels) as the overall data for serum BDNF, with the FS18+Curc treatment leading to increased BDNF from mid-point to end-point (Figure 3A). However, no significant differences were recorded for participants with normal ferritin levels. This suggests that the addition of curcumin to 18 mg iron supplementation, in particular, may be most effective at enhancing serum BDNF levels in individuals with low ferritin levels. Notably, in participants who had ‘low’ ferritin at baseline, all groups containing iron supplementation showed significant increases in ferritin values, apart from the FS18+Plac group. This indicates that the addition of curcumin alongside low dose iron supplementation may contribute to enhanced ferritin formation, which may indicate enhanced intestinal iron uptake over time. Iron status (ferritin status) can inversely influence the body’s iron absorption uptake in order to maintain homeostasis [82,83], and this may explain the lack of effect seen in participants who had ‘normal’ baseline ferritin values (Table 4). Together, these results suggest that curcumin may enhance the effects of low dose iron supplementation, in particular for those individuals with iron deficiency [77,83,84].

As iron is an essential component for mono-oxygenase enzyme production as well as a key co-factor in serotonin synthesis [20], it may be a key component of BDNF synthesis since neuroamines including norepinephrine can raise cAMP levels, leading to enhanced BDNF synthesis via increased phosphorylation of CREB [32]. Therefore, the general lack of change in BDNF levels observed overall and with participants following iron-only treatment may have occurred because iron is utilised for several biological processes following absorption, such as cellular respiration [19], and thus brain iron levels may not have been increased sufficiently to enhance neurotransmitter synthesis and BDNF expression. Compared to conventional curcumin, Briskey et al. [58] showed that a formulated curcumin form resulted in significantly greater increases in total plasma curcuminoid concentration in a human volunteer study. Furthermore, formulated curcumin has been shown to be more likely to cross the blood–brain barrier [44,54,85] and express neuroprotective effects due to improved bioavailability and targeted delivery, in both cellular and murine studies [44,85,86]. This could therefore explain how the addition of curcumin to iron supplementation consistently resulted in increased BDNF levels in the current study. There are various mechanistic pathways via which curcumin expresses its neuroprotective properties. The increase in serum BDNF observed following FS18+Curc treatment is supportive of previous studies, which have shown curcumin to increase BDNF synthesis by increasing cAMP levels and activating extracellular signal-regulated kinases (ERKs) and p38 mitogen-activated protein kinase (p38 MAPK), which are known to induce nuclear translocation of transcription factors, such as ETS-like protein-1 (Elk-1), activator protein 1(AP-1) and nuclear factor kappa B (NF_K_B) [52,87]. However, as there was an absence of a curcumin-only group in the current study, it cannot be confirmed that the observed increase in serum BDNF is solely a result of curcumin and not the result of synergic effects of the co-administration of iron and formulated curcumin supplementation. 

The current study also demonstrated an increase in serum BDNF levels between mid- and end-point in the high dose iron group (FS65+Curc), with an observable difference between FS65+Curc and FS65+Plac groups at the end-point. However, these results were not significant. This observable but non-significant change could be due to reduced fractional iron absorption (FIA); in other words, the proportion of the dose absorbed. According to Moretti et al. [88], iron doses above 60 mg, when taken on consecutive rather than alternative days, resulted in reduced FIA. This is understood to be mediated by significantly increased levels of serum hepcidin, which are observed following 24 h supplementation with ≥60 mg elemental iron [88]. Hepcidin is the key regulatory protein of systemic iron homeostasis and protects against iron overload [89]. It is synthesised mainly by the liver and regulated by iron, erythropoietic activity, hypoxia and inflammation [90]. Hepcidin regulates iron homeostasis by binding to ferroportin, the transmembrane protein that mediates iron efflux from the cells, initiating its internalisation and degradation. Consequently, the iron-efflux activity of ferroportin is down regulated, leading to a reduced dose of iron being transported to the systemic circulation from the gut enterocytes [90,91]. Stoffel et al. [92] showed that FIA was 34% lower following 14 consecutive days compared to 28 alternative days dosing with oral ferrous sulphate (60 mg elemental iron supplementation) in iron-depleted women (median age 22 years) without anaemia.

Alternatively, reduced iron absorption following consecutive days of high iron supplementation could be explained by the mucosal block theory [93]. The enterocytes uptake iron from the gut lumen via divalent metal transporter 1 (DMT1) in the ferrous (Fe^2+^) form. This iron is then converted into ferric (Fe^3+^) form and can be stored by binding to apoferritin, forming ferritin, (the temporary storage form of iron) or can be transported to the systemic circulation via ferroportin after being reduced back to its Fe^2+^ form [94]. It is assumed that enterocytes exposed to large doses of iron would store iron in ferritin form, when the body’s iron requirement is reduced. Furthermore, with the reduced availability of apoferritin, the enterocytes’ iron absorption would consequently reduce until the cells are replaced in 5 to 6 days [95] with their iron content being lost in the faeces. Nonetheless, the accuracy of mucosal block theory has been debated in recent studies [96,97,98]. However, in either case, reduced FIA or mucosal block could lead to the accumulation of large amounts of unabsorbed iron, which may cause gut inflammation [77], increase the production of free radicals in the mucosa (the innermost layer of the gut) [99,100] and may alter the gut microbiota by increasing pathogenic microorganisms whilst reducing commensal microflora [101,102]. Commensal microorganisms such as *Escherichia coli*, *Blautia sp*. (MRG-PMF1) or Lactobacillus acidophilus have been shown to be biologically active in curcumin metabolism in the gut, producing curcumin derivates with increased biological activity and improved pharmacokinetics related to absorption compared to native curcumin. Thus, microbiota may enhance the neuroprotective effects of curcumin [103]. However, the enhancing role of curcumin on gut microbiota may be diminished when compromised by high iron accumulation in the gut [104]. Additionally, the increased levels of free radicals in the gut following high iron absorption may result in the free radical scavenging properties of curcumin being utilised locally [48]. This could explain why the combination of curcumin with low iron supplementation (FS18+Curc) generally resulted in higher end-point serum BDNF levels than with high iron plus curcumin (FS65+Curc) treatment.

The normal range of serum BDNF levels varies according to numerous factors such as age, ethnicity, study location and sample processing methods [105,106,107]. However, previous research indicates that normal serum BDNF levels may fall within the range of 8 ng/mL and 46 ng/mL [108,109]. The data from the current study suggests that supplementation with 18 mg iron and 500 mg curcumin over 42 days elevates BDNF levels towards the higher end of this normal range, given the average end-point value of 39.17 ng/mL within this group (Table S1). However, a recent Swiss study of healthy adults reported a wider range of serum BDNF levels, between 15.83 ng/mL and 79.77 ng/mL [107], which highlights the difficulty in precisely establishing normal serum BDNF levels within a healthy cohort. Nevertheless, the relationship between BDNF and cognitive function is well established [110,111,112,113] and low serum BDNF levels have been attributed to cognitive impairment in the elderly [114]. Therefore, the evident increase in serum BDNF following low iron and curcumin supplementation in this cohort may be expected to be associated with probable cognitive benefits. However, in the future, it would be of value to also directly assess cognitive performance in relation to serum BDNF following iron and curcumin supplementation at varying timeframes, as this could help identify the specific levels of serum BDNF that correlate with the enhancement of cognitive function.

In summary, the current study demonstrates for the first time that the co-administration of a bioavailable formulated curcumin supplement with ferrous sulphate containing 18 mg elemental iron for 42 days results in increased serum BDNF levels. The addition of curcumin may therefore provide a novel approach to iron supplementation and possibly enhance the iron-associated cognitive benefits linked to increased serum BDNF levels.

## Figures and Tables

**Table 1:** Participant age, weight, height, body mass index (BMI) and body fat percentages (mean ± SEM).

Variable	FS0+Plac	FS18+Plac	FS18+Curc	FS65+Plac	FS65+Curc
Age (yrs)	26.29 ± 0.84	25.84 ± 0.93	24.48 ± 0.82	27.23 ± 0.83	26.77 ± 0.87
Weight (kg)	70.79 ± 2.37	72.45 ± 3.13	66.17 ± 2.45	70.21 ± 3.54	67.70 ± 2.13
Height (m)	1.72 ± 0.02	1.71 ± 0.01	1.68 ± 0.02	1.70 ± 0.02	1.72 ± 0.02
BMI (m/kg^2^)	23.89 ± 0.58	24.51 ± 0.85	23.32 ± 0.64	24.11 ± 1.00	22.83 ± 0.55
Body fat (%)	25.27 ± 1.71	24.96 ± 1.67	24.32 ± 1.76	24.97 ± 1.54	23.39 ± 1.39

**Table 2:** Participant baseline ferritin and brain-derived neurotrophic factor (BDNF) levels expressed as mean ± SEM.

Variable	FS0+Plac	FS18+Plac	FS18+Curc	FS65+Plac	FS65+Curc
Ferritin(µg/L)	58.71 ± 9.37	68.58 ± 10.30	52.36 ± 7.74	55.14 ± 8.18	61.46 ± 8.47
BDNF (ng/mL)	35.18 ± 2.10	37.16 ± 1.88	30.28 ± 1.54	31.59 ± 1.35	30.85 ± 8.47

**Table 3:** Mean ferritin (µg/L) values (low ferritin sub-group) per treatment group and timepoints (mean ± SEM). * represents significance values when comparing mid-point or end-point to baseline within the same condition (** *p* < 0.01; *** *p* < 0.001; **** *p* < 0.0001).

Timepoint	FS0+Plac	FS18+Plac	FS18+Curc	FS65+Plac	FS65+Curc
Baseline	25.08 ± 1.73	25.29 ± 2.81	30.25 ± 2.52	25.55 ± 1.78	25.04 ± 2.72
Mid-point	28.23 ± 2.96	34.59 ± 4.24	36.45 ± 2.92	36.69 ± 3.34 **	32.10 ± 4.04
End-point	31.39 ± 5.12	33.31 ± 3.39	40.89 ± 4.99 **	43.01 ± 4.01 ****	39.75 ± 6.19 ***

**Table 4:** Mean ferritin (µg/L) values (normal ferritin sub-group) per treatment group and timepoints (mean ± SEM).

Timepoint	FS0+Plac	FS18+Plac	FS18+Curc	FS65+Plac	FS65+Curc
Baseline	91.38 ± 12.94	109.17 ± 13.30	98.79 ± 15.37	94.54 ± 9.94	100.31 ± 10.01
Mid-point	93.37 ± 13.75	100.10 ± 16.85	102.23 ± 13.56	101.50 ± 11.75	101.63 ± 11.45
End-point	87.88 ± 11.45	100.21 ± 13.27	102.32 ± 16.18	98.37 ± 15.49	106.62 ± 12.29

## Supplementary Materials

The following are available online at https://www.mdpi.com/2076-3921/9/8/645/s1, Figure S1: shows the study compliance after 155 participants were enrolled and randomised equally into 5 treatment groups: FS0+Plac (full placebo, placebos for both iron and curcumin), FS18+Plac (18 mg elemental iron and placebo for curcumin), FS18+Curc (18 mg elemental iron and 500 mg curcumin), FS65+Plac (65 mg elemental iron and placebo for curcumin) and FS65+Curc (65 mg elemental iron and 500 mg curcumin). Table S1: Mean BDNF (ng/mL) values per treatment group/timepoint (mean ± SEM).
