# Supplementary material for: Co-Administration of Iron and a Bioavailable Curcumin Supplement Increases Serum BDNF Levels in Healthy Adults
Source: Antioxidants (Basel). 2020 Jul 22;9(8):645. doi: 10.3390/antiox9080645 (PMC7463477; doi:10.3390/antiox9080645)
Supplement: Supplementary file 1 [file antioxidants-09-00645-s001.pdf]

Supplementary figures

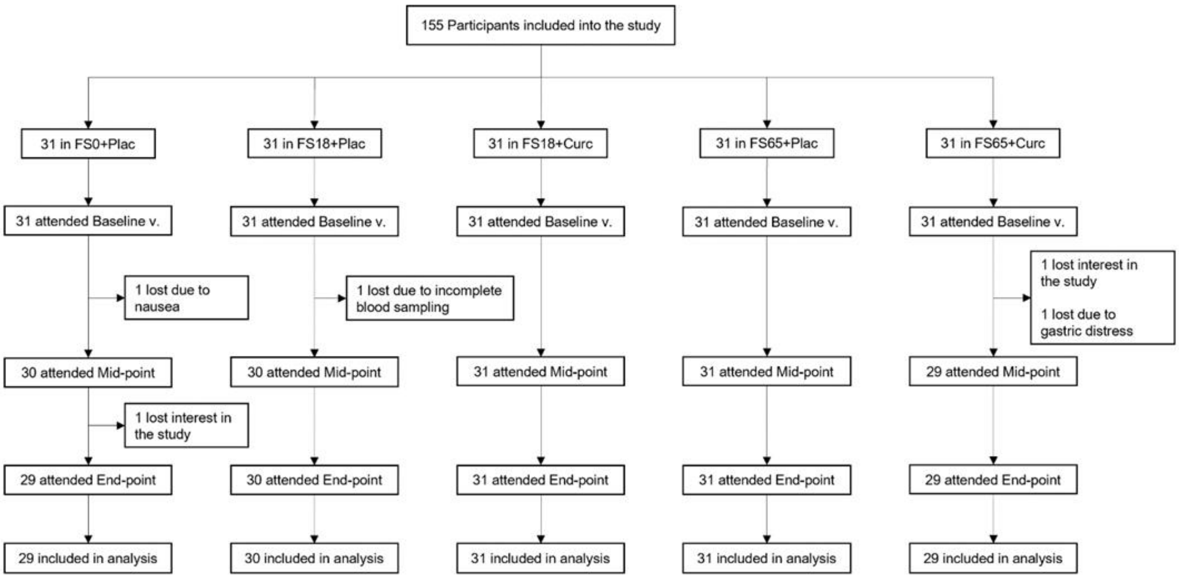

Figure S1. Study compliance after 155 participants were enrolled and randomised equally into 5 treatment groups: FS0+Plac (full placebo, placebos for both iron and curcumin), FS18+Plac (18 mg elemental iron and placebo for curcumin), FS18+Curc (18 mg elemental iron and 500 mg curcumin), FS65+Plac (65 mg elemental iron and placebo for curcumin) and FS65+Curc (65 mg elemental iron and 500 mg curcumin).

Table S1. Mean BDNF (ng/mL) values per treatment group/timepoint (mean ± SEM).

Timepoint	FS0+Plac	FS18+Plac	FS18+Curc	FS65+Plac	FS65+Curc
Baseline	35.18 ± 2.10	37.16 ± 1.88	30.28 ± 1.54	31.59 ± 1.35	30.85 ± 1.99
Mid-point	31.50 ± 1.27	32.00 ± 1.48	31.42 ± 1.02	32.64 ± 1.08	32.00 ± 1.29
End-point	36.62 ± 2.17	36.04 ± 1.66	39.17 ± 4.96	33.28 ± 1.81	39.16 ± 4.96
